# Trends in CD4 Count Testing, Retention in Pre-ART Care, and ART Initiation Rates over the First Decade of Expansion of HIV Services in Haiti

**DOI:** 10.1371/journal.pone.0146903

**Published:** 2016-02-22

**Authors:** Serena P. Koenig, Daphne Bernard, Jessy G. Dévieux, Sidney Atwood, Margaret L. McNairy, Patrice Severe, Adias Marcelin, Pierrot Julma, Alexandra Apollon, Jean W. Pape

**Affiliations:** 1 Haitian Study Group for Kaposi’s Sarcoma and Opportunistic Infections (GHESKIO), Port-au-Prince, Haiti; 2 Division of Global Health Equity, Brigham and Women’s Hospital, Harvard Medical School, Boston, MA, United States of America; 3 AIDS Prevention Program, Florida International University, Miami, FL, United States of America; 4 Center for Global Health, Weill Cornell Medical College, New York, NY, United States of America; Rega Institute for Medical Research, BELGIUM

## Abstract

**Background:**

High attrition during the period from HIV testing to antiretroviral therapy (ART) initiation is widely reported. Though treatment guidelines have changed to broaden ART eligibility and services have been widely expanded over the past decade, data on the temporal trends in pre-ART outcomes are limited; such data would be useful to guide future policy decisions.

**Methods:**

We evaluated temporal trends and predictors of retention for each step from HIV testing to ART initiation over the past decade at the GHESKIO clinic in Port-au-Prince Haiti. The 24,925 patients >17 years of age who received a positive HIV test at GHESKIO from March 1, 2003 to February 28, 2013 were included. Patients were followed until they remained in pre-ART care for one year or initiated ART.

**Results:**

24,925 patients (61% female, median age 35 years) were included, and 15,008 (60%) had blood drawn for CD4 count within 12 months of HIV testing; the trend increased over time from 36% in Year 1 to 78% in Year 10 (p<0.0001). Excluding transfers, the proportion of patients who were retained in pre-ART care or initiated ART within the first year after HIV testing was 84%, 82%, 64%, and 64%, for CD4 count strata ≤200, 201 to 350, 351 to 500, and >500 cells/mm^3^, respectively. The trend increased over time for each CD4 strata, and in Year 10, 94%, 95%, 79%, and 74% were retained in pre-ART care or initiated ART for each CD4 strata. Predictors of pre-ART attrition included male gender, low income, and low educational status. Older age and tuberculosis (TB) at HIV testing were associated with retention in care.

**Conclusions:**

The proportion of patients completing assessments for ART eligibility, remaining in pre-ART care, and initiating ART have increased over the last decade across all CD4 count strata, particularly among patients with CD4 count ≤350 cells/mm^3^. However, additional retention efforts are needed for patients with higher CD4 counts.

## Introduction

In September 2015, the World Health Organization (WHO) guidelines changed to recommend antiretroviral therapy (ART) for all HIV-infected patients, as compared to previous guidelines that recommended ART for those with a CD4 count ≤500 cells/mm^3^, ≤350 cells/mm^3^ and <200 cells/mm^3^ [1–4]. Despite these guideline changes, a substantial proportion of patients continue to initiate ART with advanced AIDS—due either to late presentation for first HIV testing, or return after loss to care. This is associated with poorer immunologic recovery and higher mortality [5–10]. Timely ART initiation requires effective pre-ART care, which includes early HIV diagnosis, effective linkage to treatment services, and retention in care until ART eligibility. Yet multiple studies have demonstrated that attrition is high at every step of pre-ART care from HIV testing to ART initiation [11–21]

Data on temporal trends in rates of completion of ART eligibility staging, pre-ART retention, and ART initiation are limited, and we are aware of no published studies that report on the change in these outcomes over the decade of ART scale-up in resource-poor settings [22–24]. We evaluated the temporal trends in CD4 count testing, retention in pre-ART care, and ART initiation rates among a cohort of adult patients from HIV testing through one year of care from 2003 to 2013 at the Haitian Study Group for Kaposi’s Sarcoma and Opportunistic Infections (GHESKIO) clinic in Port-au-Prince, Haiti.

## Methods

### Settings and Patients

Haiti is the poorest country in the Western Hemisphere, and one of the poorest in the world, ranking 168 out of 187 on the 2014 Human Development Index [25]. Haiti has the highest number of people living with HIV in the Caribbean, the region second most impacted by HIV outside of Africa. Since 1985, the dominant mode of HIV transmission in Haiti has been heterosexual intercourse. The national HIV prevalence has declined from a high of 6.2% in 1993 to 1.9% in 2014 [26].

GHESKIO is a Haitian non-governmental organization (NGO) located in Port-au-Prince, Haiti. It is the oldest and largest provider of HIV services in the Caribbean, having provided voluntary counseling and testing (VCT) for HIV since 1985. There are currently two HIV testing algorithms at GHESKIO. One utilizes the Alere Determine HIV-1/2 Ab test (Alere, Waltham, MA, USA). The other utilizes the HIV ARCHITECT HIV Ag/Ab Combo assay (Abbott Laboratories, Abbott Park, IL, USA). Positive tests are confirmed with an HIV-1/2 Ab test (Colloidal Gold, Shanghai Kehua Bio-engineering Co, Ltd, Shanghai, China). Between 2,000 and 3,000 patients receive positive HIV test results at the main GHESKIO site in downtown Port-au-Prince each year; this represents about 10% of the national total. As of December 2014, Haiti had 166 VCT sites, and 126 public and NGO-based sites provided ART [27]. At the beginning of the study period (March 2003), about 5,000 patients were receiving HIV services every year, and about 100 patients were receiving ART at the main GHESKIO site in Port-au-Prince; this increased to about 5,000 patients on ART at the end of 2008, and nearly 13,500 patients receiving HIV services in 2014, with about 9,000 on ART [27].

At GHESKIO, most patients self-present for HIV testing, rather than being referred from mobile testing centers or outside VCT clinics. Comprehensive HIV care is provided free of charge. All patients >17 years of age who received positive HIV test results between March 1, 2003 and February 28, 2013 were included in the study. Data collection continued until September 1, 2014.

### Changes in HIV Care at GHESKIO during the Study Period

HIV care at GHESKIO is provided according to the guidelines of the Haitian Ministry of Health and the WHO [1–3, 28–30]. Throughout the study period, patients were screened for tuberculosis (TB) symptoms at the time of HIV testing. Those with cough for 2 weeks or longer were screened for TB with physician evaluation, sputum microscopy, and chest radiograph. Patients diagnosed with active TB received same-day treatment when possible, and care for both diseases was provided at GHESKIO. Since 1994, isoniazid prophylaxis has been prescribed for patients without active TB who are purified protein derivative (PPD) positive [31]. Trimethoprim-sulfamethoxazole prophylaxisis is initiated for all HIV-infected patients, regardless of CD4 count, at the time that positive HIV test results are provided [32]. CD4 counts are provided on-site at GHESKIO. Patients not yet eligible for ART are scheduled for monthly visits with a clinician for the first 3 months, and then seen every 2 to 3 months. Transportation fees and phone calls for missed visits are provided to patients after they initiate ART.

During the study period, the WHO and Haitian national guidelines changed to recommend earlier treatment. Initially, ART was initiated in patients with WHO stage 4 conditions and/or CD4 count <200 cells/mm^3^. In 2009, GHESKIO clinicians began initiating ART for patients with WHO stage 3 or 4 conditions and/or CD4 count ≤350 cells/mm^3^. Of note, GHESKIO was the site of the CIPRA HT 001 study, a randomized trial that was stopped in May 2009, after demonstrating that earlier ART (CD4 count from 200 to 350 cells/mm^3^) was associated with lower mortality, compared with waiting until CD4 count <200 cells/mm^3^ or stage 4 disease [33]. In February 2014, the GHESKIO protocol again shifted (following WHO and Haitian national guidelines) to recommend ART for all patients with CD4 count ≤500 cells/mm^3^ [3, 30].

### Data Collection and Statistical Analysis

The following data were extracted from patient electronic medical records (EMRs): demographic information (age, gender, marital status, income, education, and residence zone), dates and results of HIV tests and CD4 counts, dates of initiation of TB treatment and ART, and pre-ART clinic visit dates. Data on risk factors for HIV were not captured. Patients were considered to have TB if they were given a TB diagnosis by the clinician and started on TB treatment. De-identified data were entered into an Excel database (Microsoft, Redmond, WA) and then converted to SAS version 9.2 (SAS Institute, Inc., Cary, NC). Attrition prior to CD4 count was defined as failure to have blood drawn for testing within 12 months after HIV testing, as has been recommended in other publications [34]. For each CD4 count strata (≤200, 201 to 350, 351 to 500, and >500 cells/mm^3^), we reported: the number of patients who started ART within 12 months of HIV testing; the number who remained in pre-ART care at 12 months, as defined by having at least one pre-ART visit from 9 to 15 months after HIV testing; and the number who were lost to follow up (LTFU), died, or were transferred prior to ART initiation. Due to limited resources, patient tracking activities were not conducted until patients started ART; therefore dates of transfer and death were only ascertained if patients or family members reported this information to the health care providers. Data were collected for 15 months from the time of HIV testing for each patient.

We analyzed trends over time using the Cochran-Armitage trend test, and conducted univariable and multivariable analyses of completion of blood draw for CD4 count, return for CD4 count results, and retention in pre-ART care or ART initiation for each CD4 cell strata using logistic regression. We included the following variables: gender, annual income, education, tuberculosis status at HIV testing, and residence zone (binary variables), study year and marital status (categorical variables), and age (continuous variable). For all multivariable models we included all variables that had been included in the univariable analysis. We used the Wald Confidence Interval (CI) for adjusted odds ratios, and reported 95% CIs. This study was approved by the ethics committees of GHESKIO, Weill Cornell Medical College, and Brigham and Women’s Hospital. It was not feasible to obtain informed consent for this retrospective study, but patient information was anonymized and de-identified prior to analysis.

## Results

From March 1, 2003 to February 28, 2013, 24,925 patients >17 years of age received positive HIV test results at GHESKIO. Of these, 15,115 (61%) were women, the median age was 35 years (interquartile range [IQR]: 28 to 43 years), 8,848 (35%) lived in downtown Port-au-Prince, 15,140 (62%) lived on <$US 125/year, and 14,674 (59%) had no education or primary school only; 1,491 (6%) presented with TB at HIV testing (Table 1).

**Table 1:** Baseline Characteristics of Patients Newly Diagnosed with HIV (n = 24,925).

Characteristic	Value
Female gender—no. (%)	15,115 (61)
Age—no. (%)	
18 to 24 years	3,668 (15)
25 to 34 years	8,536 (34)
35 to 44 years	7,474 (30)
≥45 years	5,247 (21)
Areas of Residence—no. (%)	
Downtown Port-au-Prince	8,848 (35)
Port-au-Prince metropolitan area other than	13,605 (55)
Downtown Port-au-Prince	
Outside of metropolitan Port-au-Prince	2,472 (10)
Annual income ≤$US 125/year—no. (%)	15,140 (62)
Education—none or primary only—no. (%)	14,674 (59)
Marital status—no. (%)	
Single	5,758 (23)
Currently married	13,119 (53)
Previously married	6,048 (24)
Tuberculosis at HIV testing—no. (%)	1,491 (6)
Baseline CD4 cell count—no. (%)*	
≤200 cells/mm^3^	5,629 (34)
201 to 350 cells/mm^3^	3,788 (23)
351 to 500 cells/mm^3^	3,109 (19)
>500 cells/mm^3^	4,143 (25)

*This includes all 16,669 patients who had blood drawn for CD4 count

**Fig 1:**
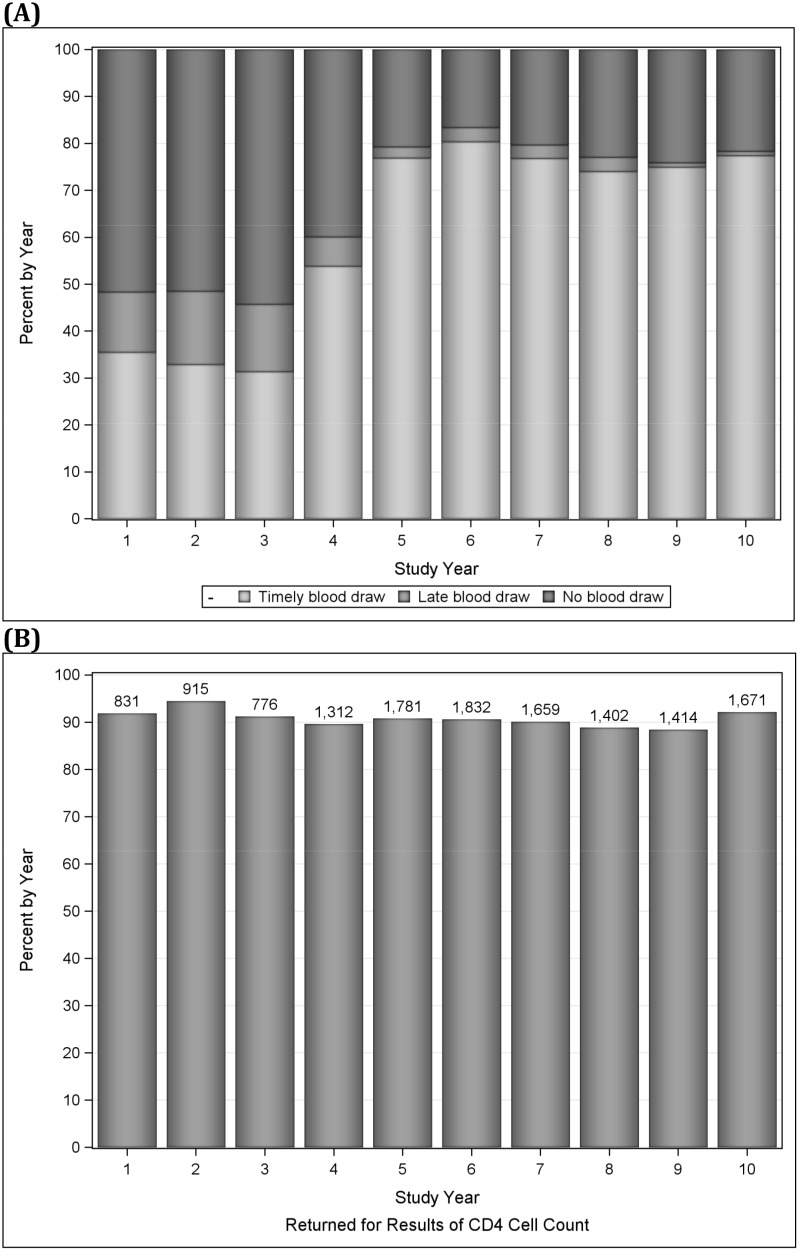
A: Proportion of Patients with Timely, Late, or No Blood Draw for CD4 Count by Year; B: Number and Proportion of Patients Returning for CD4 Count Results by Year.

Over the 10-year study period, 15,008 patients (60%) had blood drawn for a CD4 cell count within 12 months of HIV testing, and of these, 5,096 (34%), 3,405 (23%), 2,790 (19%), and 3,717 (25%) patients had a CD4 count of ≤200 cells/mm^3^, 201 to 350 cells/mm^3^, 351 to 500 cells/mm^3^, and >500 cells/mm^3^, respectively. An additional 1,661 patients had a blood draw for CD4 count more than 12 months after HIV testing, and were excluded from further analyses (Fig 1A). A total of 13,676 patients (91%) returned for CD4 count results, including 4,552 (89%), 3,147 (92%), 2,598 (93%), and 3,379 (91%) for each CD4 cell category, respectively (Fig 1B). The number of patients seeking care at GHESKIO more than doubled from Years 3 to 5; the number who returned for CD4 count test results increased from 787 to 1,802 during this period; it then decreased to 1,671 in Year 10.

The trend in having blood drawn for CD4 count within 12 months of HIV testing increased over time, from 36% in Year 1 to 78% in Year 10 (Cochran-Armitage trend test p<0.0001). The proportion returning for results remained at least 89% across all years (Fig 1A and 1B). The median time from HIV testing to CD4 count decreased from 39 days (IQR: 18 to 113 days) in Year 1 to 10 days (IQR: 7 to 18 days) in Year 10.

### Predictors of CD4 Count Completion

In multivariable analysis, factors associated with timely blood draw for CD4 count included later year of HIV test (odds ratio [OR] 1.34; 95% confidence interval [CI]: 1.33–1.36), secondary education (OR 1.37; 95% CI: 1.29–1.45), income >$US 125/year (OR 1.06; 95% CI: 1.00–1.13), older age (OR 1.22; 95% CI: 1.19–1.26), residence outside of downtown Port-au-Prince (OR 1.10; 95% CI: 1.04–1.17), and TB at HIV testing (OR 1.92; 95% CI: 1.69–2.18). Male gender (OR 0.86; 95% CI: 0.81–0.91) and single vs. married status (OR 0.91; 95% CI: 0.85–0.98) were associated with attrition prior to blood draw (Table 2).

**Table 2:** Predictors of Completing CD4 Cell Testing.

Variable	Reference Group	Predictors of Timely Blood Draw for CD4 Count				Predictors of Return for CD4 Count Results*			
		Univariable		Multivariable		Univariable		Multivariable	
		OR (95% CI)	p-value	aOR (95% CI)	p-value	OR (95% CI)	p-value	aOR (95% CI)	p-value
Study Year		1.32 (1.31–1.34)	**<0.01**	1.34 (1.33–1.36)	**<0.01**	0.96 (0.94–0.98)	**<0.01**	0.97 (0.95–0.99)	**0.01**
Secondary Education	None or Primary Only	1.19 (1.13–1.25)	**<0.01**	1.37 (1.29–1.45)	**<0.01**	1.15 (1.03–1.29)	**0.02**	1.23 (1.09–1.39)	**<0.01**
Male Gender	Female	1.02 (0.96–1.07)	0.56	0.86 (0.81–0.91)	**<0.01**	0.99 (0.88–1.11)	0.87	0.86 (0.76–0.97)	**0.02**
>$US 125/yr	≤$US 125/yr	1.38 (1.31–1.45)	**<0.01**	1.06 (1.00–1.13)	**0.04**	1.18 (1.05–1.33)	**<0.01**	1.18 (1.04–1.33)	**0.01**
Age (decade)		1.15 (1.13–1.18)	**<0.01**	1.22 (1.19–1.26)	**<0.01**	1.08 (1.03–1.14)	**<0.01**	1.09 (1.03–1.16)	**<0.01**
Marital Status									
Single	Married	0.98 (0.92–1.05)	**<0.01**	0.91 (0.85–0.98)	**<0.01**	0.89 (0.78–1.03)	0.12	0.94 (0.81–1.09)	0.81
Previously Married	Married	1.13 (1.06–1.20)	**<0.01**	1.03 (0.96–1.11)	**0.04**	0.99 (0.86–1.14)	0.51	0.92 (0.80–1.07)	0.51
Residence outside PAP**	Residence in PAP**	1.12 (1.06–1.18)	**<0.01**	1.10 (1.04–1.17)	**<0.01**	1.21 (1.08–1.36)	**<0.01**	1.16 (1.03–1.30)	**0.01**
TB at HIV Testing	No TB at HIV Testing	1.86 (1.65–2.09)	**<0.01**	1.92 (1.69–2.18)	**<0.01**	2.76 (1.99–3.82)	**<0.01**	2.91 (2.10–4.05)	**<0.01**

* The analyses of predictors of returning for CD4 count results included only patients who had blood drawn for CD4 count ≤365 days after HIV testing;

**PAP = downtown Port-au-Prince

In multivariable analysis, factors associated with returning for CD4 count results included secondary education (OR 1.23; 95% CI: 1.09–1.39), income >$US 125/year (OR 1.18; 95% CI: 1.04–1.33), older age (OR 1.09; 95% CI: 1.03–1.16), residence outside of downtown Port-au-Prince (OR 1.16; 95% CI: 1.03–1.30), and TB at HIV testing (OR 2.91; 95% CI: 2.10–4.05). Later year of HIV test (OR 0.97; 95% CI: 0.95–0.99), and male gender (OR 0.86; 95% CI: 0.76–0.97) were associated with attrition prior to returning for results (Table 2).

### Trends in Retention in Pre-ART Care and ART Initiation

**Fig 2:**
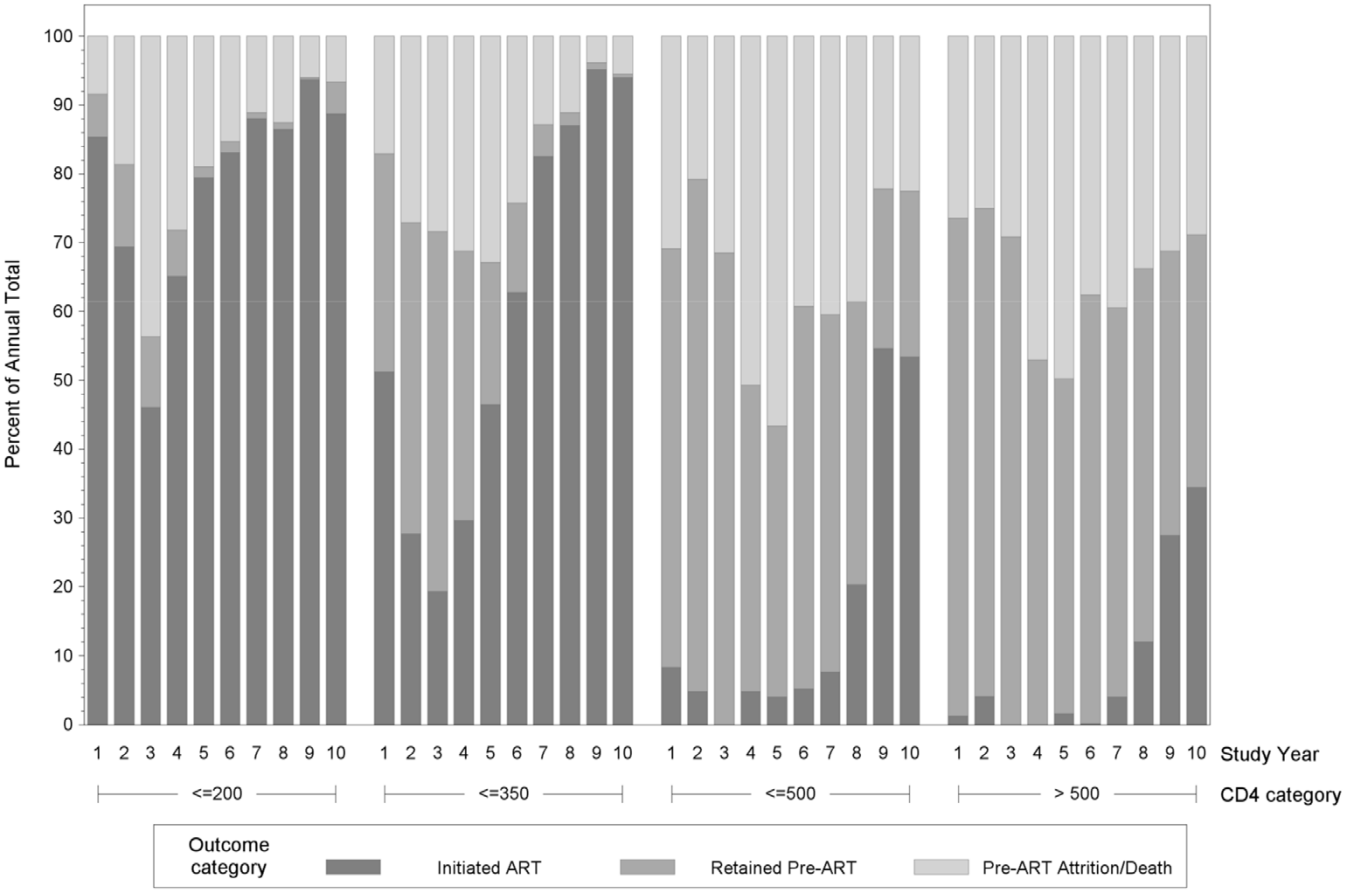
One-Year Outcomes, by Year of HIV Testing.

Among the total cohort of patients who returned for CD4 count results over the study period: 3,705 (27%) were retained in pre-ART care for at least one year; 6,257 (46%) initiated ART within the first year after HIV testing; and 3,298 (24%) were LTFU, 380 (3%) were transferred, and 36 (<1%) were known to have died during the first year after HIV testing, prior to ART initiation. The trend in retention in pre-ART care and ART initiation increased over the study period (Cochran-Armitage trend test, p<0.0001). In Year 1, 33% of patients were retained in pre-ART care, 50% initiated ART, and 17% were LTFU or dead. Outcomes initially worsened, with 28% retained in pre-ART care, 35% initiating ART, and 37% LTFU or dead in Year 5. Outcomes then improved with each subsequent year, such that in Year 10, 18% were retained in pre-ART care, 68% initiated ART, 14% were LTFU, and there were no known pre-ART deaths (Fig 2).

### Trends by CD4 Count Strata

During the study period, 4,552 patients had a baseline CD4 count ≤200 cells/mm^3^, and of these, 270 (6%) were retained in pre-ART care and 3,484 (77%) initiated ART within the year after HIV testing. Six hundred eighty-three patients (15%) were LTFU, 95 (2%) were transferred, and 20 (<1%) died prior to ART initiation. The trend in retention in pre-ART care and ART initiation increased over the study period (Cochran-Armitage trend test, p<0.0001). In Year 10, 5% were retained in pre-ART care, 89% initiated ART, 6% were LTFU, and none were known to have died prior to initiating ART (Fig 2). The median time from CD4 count to ART initiation among patients with CD4 count ≤200 cells/mm^3^ was 13 days (IQR: 5 to 28 days) in Year 1, and 11 days (IQR: 7 to 17 days) in Year 10.

A total of 3,147 patients had a baseline CD4 count between 201 and 350 cells/mm^3^, and among these, 538 (17%) were retained in pre-ART care and 1,977 (63%) initiated ART within the first year after HIV testing. Five hundred fifty-six patients (18%) were LTFU, 66 (2%) were transferred, and 10 (<1%) died prior to ART initiation. The trend in retention in pre-ART care or ART initiation increased over the study period (Cochran-Armitage trend test, p<0.0001). In Year 10, 1% of these patients were retained in pre-ART care, 94% initiated ART, 5% were LTFU, and none were known to have died prior to ART initiation (Fig 2).

A total of 2,598 patients had a baseline CD4 count between 351 and 500 cells/mm^3^, and among these, 1,137 (44%) were retained in pre-ART care and 460 (18%) initiated ART within the first year after HIV testing. Eight hundred ninety-eight patients (35%) were LTFU, 100 (4%) were transferred, and 3 (<1%) died prior to ART initiation. The trend in retention in pre-ART care or ART initiation increased over the study period (Cochran-Armitage trend test, p<0.0001). In Year 10, 26% were retained in pre-ART care, 53% initiated ART, 21% were LTFU, and none were known to have died prior to initiating ART (Fig 2).

A total of 3,379 patients had a baseline CD4 cell count >500 cells/mm^3^, and of these, 1760 (52%) were retained in pre-ART care and 336 (10%) initiated ART within the first year after HIV testing, One thousand one hundred sixty-one patients (34%) were LTFU, 119 (4%) were transferred, and 3 (<1%) died prior to ART initiation. The trend in retention in pre-ART care or ART initiation increased over the study period (Cochran-Armitage trend test, p = 0.0051). In Year 10, 40% of patients were retained in pre-ART care, 34% initiated ART, 26% were LTFU, and there were no known pre-ART deaths (Fig 2).

### Predictors of Pre-ART Retention and ART Initiation

In multivariable analyses (Tables 3 and 4), later year of HIV test was associated with retention in care for patients in all CD4 count strata: CD4 count ≤200 cells/mm^3^ (OR 1.16; 95% CI: 1.12–1.19); 201 to 350 cells/mm^3^ (OR 1.24; 95% CI: 1.19–1.29); 351 to 500 cells/mm^3^ (OR 1.08; 95% CI: 1.05–1.12); and >500 cells/mm^3^ (OR 1.05; 95% CI: 1.02–1.08). Secondary education was also associated with retention in care for all CD4 strata: CD4 count ≤200 cells/mm^3^ (OR 1.54; 95% CI: 1.29–1.84); 201 to 350 cells/mm^3^ (OR 1.27; 95% CI: 1.04–1.55); 351 to 500 cells/mm^3^ (OR 1.48; 95% CI: 1.24–1.77); and >500 cells/mm^3^ (OR 1.46; 95% CI: 1.25–1.70). Among patients with low CD4 counts, income >$US 125/year was associated with retention in care: CD4 count ≤200 cells/mm^3^ (OR 1.33; 95% CI: 1.11–1.59) and 201 to 350 cells/mm^3^ (OR 1.30; 95% CI: 1.06–1.58). Among patients with high CD4 counts, male gender was associated with pre-ART attrition: CD4 count 351 to 500 cells/mm^3^ (OR 0.78; 95% CI: 0.65–0.93) and CD4 count >500 cells/mm^3^ (OR 0.75; 95% CI: 0.63–0.88). TB at HIV testing was associated with pre-ART attrition for patients with CD4 count ≤200 cells/mm^3^ (OR 0.61; 95% CI: 0.48–0.77), but with improved retention in care for patients with higher CD4 counts: CD4 count 351 to 500 cells/mm^3^ (OR 2.09; 95% CI: 1.43–3.05) and CD4 count >500 cells/mm^3^ (OR 2.37; 95% CI: 1.59–3.52).

**Table 3:** Predictors of Remaining in Pre-ART Care or Initiating ART among Patients with CD4 Count ≤350 cells/mm^3^.

Variable	Reference Group	CD4 Cell Count ≤200 Cells/mm^3^				CD4 Cell Count 201 to 350 Cells/mm^3^			
		Univariable		Multivariable		Univariable		Multivariable	
		OR (95% CI)	p-value	aOR (95% CI)	p-value	OR (95% CI)	p-value	aOR (95% CI)	p-value
Study Year		1.18 (1.14–1.21)	**<0.01**	1.16 (1.12–1.19)	**<0.01**	1.24 (1.20–1.29)	**<0.01**	1.24 (1.19–1.29)	**<0.01**
Secondary Education	None or Primary Only	1.69 (1.43–2.00)	**<0.01**	1.54 (1.29–1.84)	**<0.01**	1.31 (1.09–1.58)	**<0.01**	1.27 (1.04–1.55)	**0.02**
Male Gender	Female	1.03 (0.87–1.21)	0.75	0.93 (0.78–1.11)	0.42	1.04 (0.86–1.25)	0.70	0.96 (0.78–1.17)	0.68
>$US 125/yr	≤$US 125/yr	1.53 (1.28–1.83)	**<0.01**	1.33 (1.11–1.59)	**<0.01**	1.56 (1.28–1.89)	**<0.01**	1.30 (1.06–1.58)	**0.01**
Age (decade)		1.02 (0.94–1.10)	0.69	1.04 (0.95–1.13)	0.41	1.06 (0.98–1.15)	0.16	1.13 (1.02–1.25)	**0.02**
Marital Status									
Single	Married	1.12 (0.91–1.39)	0.29	1.13 (0.90–1.43)	0.42	1.04 (0.82–1.31)	0.68	1.07 (0.84–1.37)	0.50
Previously Married	Married	1.01 (0.84–1.22)	0.61	1.07 (0.87–1.31)	0.98	0.98 (0.78–1.22)	0.70	0.97 (0.76–1.22)	0.58
Residence outside PAP*	PAP*	0.97 (0.81–1.16)	0.73	0.94 (0.79–1.13)	0.51	0.88 (0.72–1.06)	0.18	0.90 (0.74–1.10)	0.31
TB at HIV Testing	No TB at HIV Testing	0.63 (0.50–0.80)	**<0.01**	0.61 (0.48–0.77)	**<0.01**	0.82 (0.60–1.12)	0.22	0.88 (0.63–1.22)	0.44

*PAP is residence in downtown Port-au-Prince

**Table 4:** Predictors of Remaining in Pre-ART Care or Initiating ART among Patients with CD4 Count >350 cells/mm^3^.

Variable	Reference Group	CD4 Cell Count 351 to 500 Cells/mm^3^				CD4 Cell Count >500 Cells/mm^3^			
		Univariable		Multivariable		Univariable		Multivariable	
		OR (95% CI)	p-value	aOR (95% CI)	p-value	OR (95% CI)	p-value	aOR (95% CI)	p-value
Study Year		1.08 (1.04–1.11)	**<0.01**	1.08 (1.05–1.12)	**<0.01**	1.04 (1.01–1.07)	**0.01**	1.05 (1.02–1.08)	**<0.01**
Secondary Education	None or Primary Only	1.44 (1.22–1.70)	**<0.01**	1.48 (1.24–1.77)	**<0.01**	1.37 (1.19–1.59)	**<0.01**	1.46 (1.25–1.70)	**<0.01**
Male Gender	Female	0.90 (0.76–1.07)	0.22	0.78 (0.65–0.93)	**0.0051**	0.86 (0.73–1.01)	0.06	0.75 (0.63–0.88)	**<0.01**
>$US 125/yr	≤$US 125/yr	1.22 (1.03–1.44)	**0.02**	1.17 (0.98–1.39)	0.0752	1.04 (0.90–1.21)	0.58	0.98 (0.84–1.14)	0.75
Age (decade)		1.06 (0.99–1.14)	0.09	1.12 (1.03–1.21)	**0.0087**	1.03 (0.97–1.09)	0.42	1.10 (1.02–1.18)	**0.01**
Marital Status									
Single	Married	0.94 (0.77–1.14)	0.62	0.96 (0.77–1.18)	0.9307	0.84 (0.71–1.00)	0.17	0.78 (0.65–0.94)	0.16
Previously Married	Married	0.96 (0.79–1.18)	0.97	0.90 (0.73–1.11)	0.4406	0.90 (0.75–1.08)	0.81	0.80 (0.66–0.97)	0.30
Residence outside PAP*	PAP*	1.14 (0.96–1.35)	0.14	1.16 (0.98–1.37)	0.0889	0.99 (0.85–1.14)	0.85	0.95 (0.82–1.09)	0.45
TB at HIV Testing	No TB at HIV Testing	1.97 (1.34–2.91)	**<0.01**	2.09 (1.43–3.05)	**0.0001**	2.05 (1.38–3.03)	**<0.01**	2.37 (1.59–3.52)	**<0.01**

*PAP is residence in downtown Port-au-Prince

## Discussion

We found that completion rates for multiple steps of pre-ART care including assessment for ART eligibility with CD4 count testing, pre-ART retention, and ART initiation have improved over the last decade at the largest HIV clinic in the Caribbean. The proportion of patients having blood drawn for CD4 count within 12 months of HIV testing increased from 36% in Year 1 to 78% in Year 10. We attribute this to a decrease in the median time from HIV testing to CD4 count (39 days in Year 1 to 10 days in Year 10). This was accomplished by implementing a policy to complete CD4 count testing as quickly as possible; CD4 counts are generally ordered the first time an HIV-infected patient sees a GHESKIO physician. Pre-ART retention and ART initiation rates improved over time for patients in all CD4 count strata. Outcomes improved with each subsequent year, except for Years 3 to 5, when the annual number of patients completing CD4 counts more than doubled, and retention temporarily declined. After Year 5, rates of retention and ART initiation progressively improved, and in the Year 10 cohort, only 14% of patients were LTFU.

ART initiation rates were highest among patients with CD4 count ≤350 cells/mm^3^. In Year 10, 89% of patients with CD4 count ≤200 cells/mm^3^ and 94% with CD4 count from 201 to 350 cells/mm^3^ initiated ART within one year of HIV testing. This is higher than ART initiation rates reported from many HIV programs in resource-poor settings. A systematic review from Africa found a median of 68% (range 14 to 84%) of patients who qualified for ART initiated treatment, and a meta-analysis found that 63% of qualifying patients in African studies initiated therapy [16, 17]. For patients with CD4 counts from 201 to 350 cells/mm^3^ in particular, we attribute the increase in ART initiation rates in part to the change in treatment guidelines, implemented at GHESKIO during Year 7, which made these patients eligible for ART.

Though it improved over time, retention remained lowest among patients with CD4 count >350 cells/mm^3^. In Year 10, 21% of patients with CD4 count from 350 to 500 cells/mm^3^ and 26% with CD4 count >500 cells/mm^3^ were LTFU. The majority of studies from other resource-poor settings document even higher rates of pre-ART attrition in patients with early HIV disease. A systematic review from Africa found a median of 46% (range 31 to 95%) of patients not yet eligible for ART were LTFU during the pre-ART period [16]. In Johannesburg, 74% of patients with CD4 count >350 cells/mm^3^ did not return within one year for a first medical visit, and only 50% of patients not eligible for ART repeated their CD4 count within the subsequent year [12, 35]. In KwaZulu-Natal only 43% of patients with CD4 count from 351 to 500 cells/mm^3^ and 35% with CD4 cell count >500 cells/mm^3^ had a repeat CD4 count within the subsequent 13 months [15]. In Cape Town, 46% of patients not yet eligible for ART returned for a repeat CD4 count after a median time of 8 months [13]. In Cambodia, 60% of patients who didn’t qualify for ART at HIV testing remained in care for one year, compared to 92% of those who initiated ART [36].

Possible explanations for the higher retention rate observed at GHESKIO include more frequent visits and a dedicated pre-ART clinic that opened in 2012. Pre-ART patients are seen monthly for the first quarter, and then at least quarterly. Patients also receive prophylactic medications, including isoniazid and trimethroprim-sulfamethoxazole, regardless of CD4 count, which was found to improve pre-ART retention in care in Africa [37]. Even so, pre-ART retention rates for patients with early HIV disease at GHESKIO remain suboptimal. As in many other HIV programs, GHESKIO does not provide transportation subsidies for patients in pre-ART care [18, 24, 38, 39]. Though resources are very limited, financial enablers may improve retention in care [40–43]. Furthermore, in most countries, patients with CD4 count >500 cells/mm^3^ do not meet immunologic criteria for ART initiation [3]. As countries adopt the recently updated WHO guidelines, which recommend treatment for all HIV-infected patients, the ART initiation rate for this cohort will likely increase, with associated reductions in pre-ART attrition [4].

Throughout the study period, TB at HIV testing was associated with completion of CD4 count, and with retention in care for patients with CD4 counts >350 cells/mm^3^. We attribute this to fast tracked services, as patients with TB symptoms at HIV testing receive same-day TB testing, and when possible, same-day TB treatment as well. Since HIV and TB treatment services are integrated at GHESKIO, clinicians provide HIV services at the same time they provide TB care. For patients with CD4 count ≤200 cells/mm^3^, TB at HIV testing was associated with attrition. We attribute this to a higher risk of death in these patients.

Residence outside of downtown Port-au-Prince (where GHESKIO is located) was associated with completion of CD4 count testing, but not with retention in care among those who completed testing. We attribute this in part to the desire of some patients who live near GHESKIO to receive care away from their homes, once they have found out that they are infected, to avoid being recognized; HIV is a stigmatized disease in Haiti. It is also probable that patients who come from farther away have made a special trip to the clinic, so are more likely to complete their lab testing.

Male gender is associated with late HIV testing and more advanced disease at ART initiation [44–51], which is strongly associated with poorer outcomes [33]. We found that males were less likely to complete CD4 cell testing, and that among patients with CD4 count >350 cells/mm^3^, males were less likely to remain in pre-ART care or initiate ART. Studies from other sites have found conflicting results; reports from Mozambique, Nigeria and Kenya [22, 24, 38, 52] detected no association, but studies from Uganda, Malawi and South Africa found that male gender was associated with pre-ART attrition [15, 38, 42]. Innovative outreach efforts for earlier HIV testing and timely ART initiation for males are critical to maximize HIV treatment outcomes.

A major strength of this study is that we include trends in rates of retention in pre-ART care and ART initiation over the decade of expansion of access to ART services. Though our study was conducted at the largest HIV clinic in the Caribbean, the results may not be generalizable to other HIV clinics in resource-poor settings. Our study was also limited by the inability to determine vital status for patients who were LTFU, though in a prior study that included patient tracking, we found that only 12% of patients lost prior to ART initiation had received care in a different HIV clinic [53].

## Conclusions

In summary, completion rates for multiple steps in pre-ART care including assessment of ART eligibility with CD4 count testing, pre-ART retention, and ART initiation have improved over the last decade across all CD4 strata, and are particularly high among patients with CD4 count ≤350 cells/mm^3^. However, additional retention efforts are needed to help patients with higher CD4 counts, males, younger patients, and patients with poor socio-economic status to remain in care. Implementation of the recent WHO recommendation for universal treatment may improve retention among patients with early HIV disease.
